# The effects of multi-generational exposure to Fluorine-Free Foam (F3) alternatives on *Daphnia magna* tolerance

**DOI:** 10.1007/s10646-025-02938-0

**Published:** 2025-07-16

**Authors:** Jack D. Morehouse, Devin K. Jones, Jason T. Hoverman

**Affiliations:** 1 Purdue University, Department of Biological Sciences, West Lafayette, IN USA; 2 Purdue University, Department of Forestry and Natural Resources, West Lafayette, IN USA

**Keywords:** Contaminants of concern, Adaptation, Non-monotonic response, Sublethal effects

## Abstract

Per- and polyfluoroalkyl substances (PFAS) have historically been a key component in aqueous film-forming foams (AFFF) used in fire suppression. With the increasing emphasis on phasing out PFAS use due to health and environmental concerns, several new chemical technologies have been used to create fluorine-free foam (F3) alternatives. Recent research has demonstrated that these replacement formulations are more acutely toxic to aquatic species than the traditional PFAS-containing AFFF. Given their relatively high acute toxicity, prolonged exposure to the formulations could lead to adaptive responses (i.e., evolved tolerance) in exposed populations. In this study, we examined the effects of chronic exposure to six F3 alternatives and one PFAS-containing AFFF on tolerance to the formulations in the water flea *Daphnia magna*. Following an 84-day exposure to different concentrations of each formulation, we used a series of laboratory lethal concentration (LC_50_) tests on a subset of populations to examine the potential change in tolerance. We found that chronic exposure to three formulations led to a change in tolerance in exposed populations as compared to those with no prior exposure; two populations displayed increased tolerance while one showed decreased tolerance. This work is the first to examine evolved responses to F3 alternatives. Our results highlight the frequently overlooked evolutionary effects of contaminant exposure and the broader need to understand the effects of F3 alternatives on the environment.

## Introduction

Aqueous film-forming foams (AFFF) containing per- and polyfluoroalkyl substances (PFAS) have played a crucial role in extinguishing hydrocarbon fuel fires (Hetzer et al. 2014). As such, AFFF have been used for decades at military and municipal facilities where flammable hydrocarbons pose risks to human life and infrastructure (Moody and Field, 2000). However, AFFF runoff into habitats adjacent to or downgradient from these application sites can introduce AFFF constituents into aquatic ecosystems (Baduel et al. 2015; Sharifan et al. 2021). Field studies assessing PFAS, a key constituent in AFFF, frequently detect high levels of PFAS in aquatic ecosystems in close proximity to AFFF application sites (Sims et al. 2022). Given the chemical stability imparted by the strength of carbon-fluorine bonds present in PFAS, there is limited environmental degradation. Moreover, PFAS tend to bioaccumulate (Sunderland et al. 2019) and biomagnify in contaminated systems (Giesy and Kannan, 2001; Haukås et al. 2007; Sharifan et al. 2021), which is concerning because PFAS exposure can affect the physiology and development of wildlife and humans (Ankley et al. 2021; Sinclair et al. 2020; Tornabene et al. 2021).

Due to these concerns, there has been an emphasis on developing fluorine-free foam (F3) alternatives, which do not contain PFAS, to preserve the vital fire-suppressing function of traditional PFAS-containing AFFF. As a part of this effort, through the 2020 National Defense Authorization Act (NDAA, 2019), the United States Congress directed the Department of Defense (DoD) to cease usage of AFFF and begin using F3 alternatives by October 2024. To comply with this mandate, in 2020 the DoD selected six F3 formulations as potential replacements for the currently used PFAS containing AFFF Buckeye Platinum Plus C6MILSPEC 3%. The F3 alternatives were National Foam Avio F3 Green KHC 3% (Avio), Bio-Ex ECOPOL A 3% FFF (ECOPOL), FOMTEC Enviro 2–3% FFF (FOMTEC), Solberg Re-Healing Foam RF3 3% (ReHealing), National Foam NFD 20-391 (NFD), and Naval Research Laboratory 502W (502W). These new formulations employ and often combine a wide range of technologies, such as alternative hydrocarbon surfactants, silicon-based surfactants, microspheres, and ionic liquids (Ateia et al. 2023), while containing less than 1 ppb PFAS in the formulation, per DoD specification (MIL-PRF-32725). Due to the recent development of these foams, very little F3 application has occurred. While this limits our ability to project the expected environmental concentration of F3 alternatives in aquatic habitats, it is assumed that F3 application procedure and frequency will be comparable to the applications of their AFFF predecessors (Ateia et al. 2023). The novel nature of these formulated products and the uncertainties surrounding fate and transport leave knowledge gaps that would typically inform ecotoxicological studies. Despite this challenge, studies investigating F3 formulated products have the unique opportunity to investigate the toxic effects of contaminants of concern before they enter ecosystems.

In addition to assessing the functional efficacy of candidate F3 alternatives, the DoD’s Strategic Environmental Research and Development Program (SERDP) funded a working group to determine the toxicity of F3s to freshwater and marine species and to characterize the relative toxicity of F3s compared to PFAS-containing AFFF formulations. The work of this group found that all of the F3 alternatives were just as, if not more, acutely toxic across all tested taxa than the PFAS-containing AFFF they would be replacing (Holden et al. 2023; Jones et al. 2022). However, it remains unknown how wildlife populations respond to chronic exposure to F3 alternatives at concentrations below those that can cause direct mortality.

Exposure to synthetic chemicals can increase selective pressure on wildlife, resulting in rapid adaptation in exposed populations (Ahmed and Freed, 2021; Hua et al. 2015). For example, *Daphnia magna* (water fleas) have displayed an ability to rapidly evolve tolerance to pesticides as well as metals (Jansen et al. 2015; Ward and Robinson, 2005). Given the acute toxicity of some F3 alternatives, chronic exposure over multiple generations could place a strong selective pressure on populations and favor the development of increased tolerance. We sought to examine the potential evolutionary effects of chronic F3 alternative exposure on *D. magna* tolerance. *Daphnia* are prey for a variety of macroinvertebrates, larval amphibians, and fish species and maintain water quality and clarity through the consumption of phytoplankton (Berga et al. 2015; Lathrop et al. 1999; Sarnelle, 2005). Moreover, their ubiquitous range, short generation time, and ease of experimentation make *D. magna* an ideal candidate for studies investigating the long-term effects of novel contaminants in aquatic systems. We exposed populations of *D. magna* to different concentrations of seven formulations, including one AFFF and six F3 alternatives, for 84 days under controlled conditions before testing their tolerance using lethal concentration (LC_50_) tests. We hypothesized that populations exposed to the formulations would exhibit increased tolerance to the formulations relative to unexposed populations. We also hypothesized that tolerance within exposed populations would increase with formulation concentration in a dose-dependent fashion due to increased strength of selection.

## Materials and methods

### Overview

**Fig. 1:**
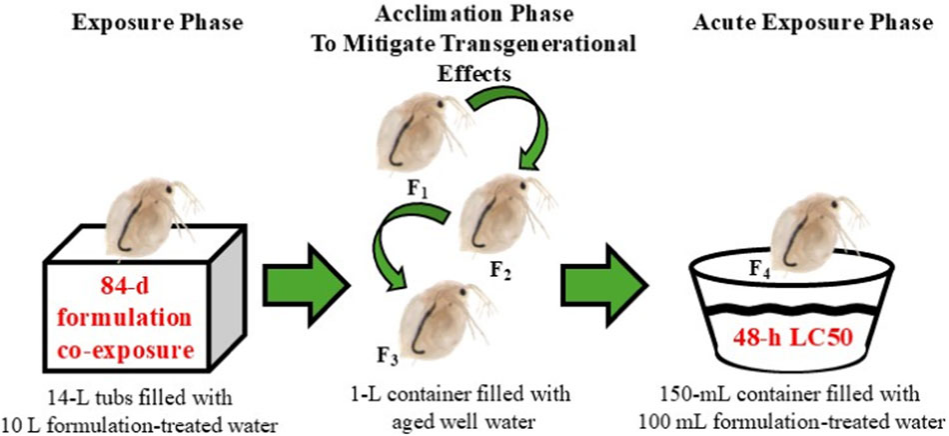
Schematic diagram showing experimental design consisting of exposure, acclimation, and acute exposure phases

We examined the potential effects of chronic exposure to the formulations on the survival and tolerance of *D. magna* using a series of lethal concentration tests at Purdue University’s Wildlife Ecology Research Facility (West Lafayette, IN, USA). To address our hypotheses, we tested a subset of exposed zooplankton populations following an 84-d chronic exposure to one of six F3 alternatives or one AFFF formulations (Fig. 1). Zooplankton were collected opportunistically from a laboratory experiment conducted with gray treefrog tadpoles (*Hyla versicolor*) in the summer of 2022. Further details regarding this experiment are viewable in Hoverman et al. (2025). The *D. magna* were included in the experiment to help maintain water quality during the exposure. The experiment concurrently exposed gray treefrogs and *D. magna* to one of six concentrations of the seven formulations for 84 days. For each formulation, the selected exposure concentrations were based on previously reported lethal concentration values for gray treefrogs (Jones et al. 2022). Each treatment was replicated four times for 24 total experimental units per formulation and 168 experimental units in total. We collected populations of zooplankton from replicates in the non-exposure (i.e., control), low, and high treatments of each formulation. In total, we were able to recover and test for evolved tolerance in zooplankton populations from 22 treatments (Table 1).

**Table 1:** Exposure information for the 22 tested *D. magna* populations

Formulation	Mean dose (mg/L)	Number of tests
Avio Green	0.0	6
	0.0533	2
	4.96	1
ECOPOL A	0.0	3
	0.090	2
	17.02	3
FOMTEC Enviro	0.0	3
	0.1542	6
	30.33	1
NFD 20-391	0.0	4
	0.1112	2
	19.77	2
NRL 502W	0.0	2
	7.55	4
	150.63	2
Solberg Re-Healing	0.0	4
	0.1264	4
	88.24	4
Buckeye Platinum	0.0	3
	2.5442	3
	252.54	5
	500.05	2

‘Number of tests’ refers to the number of lethal concentration tests conducted using each *D. magna* population

### Chemical background

Buckeye Platinum Plus C6 MILSPEC 3% (Buckeye) was the PFAS-containing reference formulation. Buckeye was certified for military use and approved on the DOD’s Qualified Products Listing in 2004. Buckeye contained 3.75 g/L 6:2 fluorotelomer sulfonate (FTS) and 0.332 g/L perfluorohexanoic acid (PFHxA) as well as a 6:2 fluorotelomer zwitterion surfactant identified as C_16_H_23_F_13_N_2_O_6_S_2_. Further analytical details and tentative structures of the identified fluorosurfactant are described in Jones et al. (2022).

We selected National Foam Avio F3 Green KHC 3% (Avio), Bio-Ex ECOPOL A 3% FFF (ECOPOL), FOMTEC Enviro 2–3% FFF (FOMTEC), Solberg Re-Healing Foam RF3 3% (ReHealing), National Foam NFD 20-391 (NFD), and Naval Research Laboratory 502W (502W) as our F3 alternative formulations. These formulations were being considered by the Department of Defense (DOD) Strategic Environmental Research and Development Program (SERDP) as potential replacements for PFAS-containing AFFF. The formulations were provided to us by SERDP for the experiments. We attempted to verify exposure concentrations (Table 1) using a semi-quantification approach. Results of this analysis are available in the supplementary information of Hoverman et al. (2025). Currently, little is known about the persistence, fate, and transport of F3 formulations and analytical chemistry methods remain nascent (Jahura et al. 2024). Exacerbated by the mixture status of the F3 alternatives, measuring dosing levels for acute toxicity assays was not pragmatic.

### Exposure phase

On March 3, 2022, we placed 300 free-swimming tadpoles and roughly 50 adult zooplankton [clonal culture of D. magna originally obtained from Aquatic BioSystems, Inc. (Fort Collins, CO, USA)] reared on laboratory cultured *Raphidocelis subcapitata*) in common density outdoor mesocosms filled with 150 L of aged well water. Tadpoles and zooplankton were fed Purina® Rabbit Complete pellets (Arden Hills, MN, USA) *ad libitum* and received full water exchanges as needed. Each mesocosm was covered with a 60% shade cloth to simulate forest canopy, moderate temperature, and exclude predators.

Three weeks later, all tadpoles were collected from each common density mesocosm and combined before 15 tadpoles were distributed in each ethanol pre-cleaned 14-L Sterilite® experimental unit. Zooplankton were simultaneously stocked in each experimental unit with each group of 15 gray treefrog tadpoles. Every four days, gray treefrog tadpoles and zooplankton were transferred into duplicate replicates to conduct a full water exchange and formulation renewal. Following every water exchange, each experimental unit received an agar-based food (ground TetraMin® Tropical Flakes [Tetra, Blacksburg, Virginia], ground rabbit chow, and agar [Fisher Scientific; CAS #9002-18-0) that equaled 40% of the surviving gray treefrog biomass. Each experimental unit was then completely emptied and dried before being refilled (10 L) and dosed with the appropriate formulation concentration two days prior to the next water exchange. Dosed treatments were permitted 48 h to fully homogenize prior to the addition of tadpoles and zooplankton. Gray treefrog tadpoles and zooplankton were exposed to each formulation-by-concentration treatment for a total of 84 days (Fig. 1).

### Acclimation phase

At the conclusion of the exposure phase (August 22, 2022), we collected zooplankton from the exposure units (Table 1) and homogenized each as a formulation-by-concentration population in 1-L plastic containers filled with 0.9 L of aged well water. Zooplankton from each dose of each formulation were treated as, and will henceforth be referred to as, discrete populations. Zooplankton were fed *ad libitum* cultured *R. subcapitata* (3 × 10^7^ cells mL^−1^) and monitored for reproduction. For each treatment population, we discarded individuals from the first reproductive event (defined as any offspring produced in a three-day period following the initial observation of a single offspring) and randomly selected 10 offspring in a subsequent reproductive event to be placed in 1-L containers filled with 0.9 L of aged well water. We continued this reproductive procedure until four total generations were collected for each population (Fig. 1). Previous literature has shown that food availability in previous generations can impact *D. magna* life history traits via maternal effects (Garbutt et al. 2014). Additionally, maternal effects can impact fitness in the generation immediately following contaminant exposure (Castro et al. 2018). We sought to eliminate these confounding effects using an acclimation procedure, as is common in *Daphnia* studies. After the initial reproductive event of the fourth generation was discarded, we collected, enumerated, and housed offspring from each subsequent reproductive event (reproductive output over 3 days) in ethanol pre-cleaned Sterilite® 14-L containers filled with 10 L of aged well water. Individuals developed under laboratory conditions (22 °C; 16:8 h light:dark cycle) for 7–10 days and were fed *R. subcapitata ad libitum* daily prior to exposure in lethal concentration tests. Ephippia (resting eggs) were not observed at any point during the acclimation phase. While the clutch number used for lethal concentration tests varied among populations, age of offspring (7–10 d) in the lethal concentration tests remained consistent among all populations.

### Acute exposure phase

We assessed zooplankton tolerance to the formulations using lethal concentration tests (LC_50_). As done in previous F3 toxicity tests, individuals were collected over a 3-day reproductive period and reared between 7 and 10 days of age for each LC_50_ test (Jones et al. 2022). For each test concentration, we massed each formulation in 60-mL plastic cups that were then submerged in 1-L containers filled with 0.9 L of aged well water two days prior to the start of testing to promote chemical homogenization. On the second day after dosing, we then distributed approximately 100 mL of the gently homogenized formulation-exposed water to each replicate. Experimental units were ethanol pre-cleaned 150-mL plastic containers. Once filled, each experimental unit received a single zooplankton added using a Fisherbrand® transfer pipette. We transferred individuals in a single drop of water to reduce dilution of the testing solution and to avoid air infiltration beneath the carapace. Zooplankton mortality was observed every 24 h for 48 h following addition. We assessed mortality as the absence of independent movement after gentle agitation with pulses of water from a disposable transfer pipette. Zooplankton were not fed during the 48-h lethal concentration exposure. Laboratory conditions during all LC tests were identical to the acclimation phase (22 °C; 16:8 h light:dark cycle). If the selected dose regime was insufficient to induce mortality during the 48-h exposure, the test for that respective population was repeated with a higher dose regime. Doses used for each chemical ranged as follows: Avio 10–300 mg/L; ECOPOL 10–400 mg/L; NFD 10–200 mg/L; FOMTEC 25–320 mg/L; Re-Healing 40–600 mg/L; 502W 500–3000 mg/L; Buckeye 1000–4250 mg/L.

### Statistical methods

We calculated lethal concentration values to assess changes in the tolerance of populations following formulation exposure. Using mortality data collected following 48 h of exposure, we examined the LC_10_, LC_50_, and LC_90_ values for each formulation-by-concentration population. The LC_10_, LC_50_, and LC_90_ values describe the concentration of a contaminant that is expected to cause 10, 50, or 90% mortality of a population, respectively, following an exposure. We employed the *ecotox* package of R (R Development Core Team, 2024) and used the ‘LC_logit’ function to calculate the LC_10_, LC_50_, and LC_90_ values using logit models. To compare LC_50_ values between zooplankton populations, we used the ratio test (‘ratio_test’ function in the *ecotox* package) developed by Wheeler et al. 2006. All test output was interpreted at α = 0.05.

## Results

Of the formulations tested, we found significant changes in *D. magna* tolerance with three formulations (Avio Green, ECOPOL A, and NFD 20-391), marginal changes with one formulation (FOMTEC Enviro), and no change with three formulations (NRL 502W, Solberg Re-Healing, Buckeye). Below, we discuss the results for the four significant and marginally significant formulations.

**Fig. 2:**
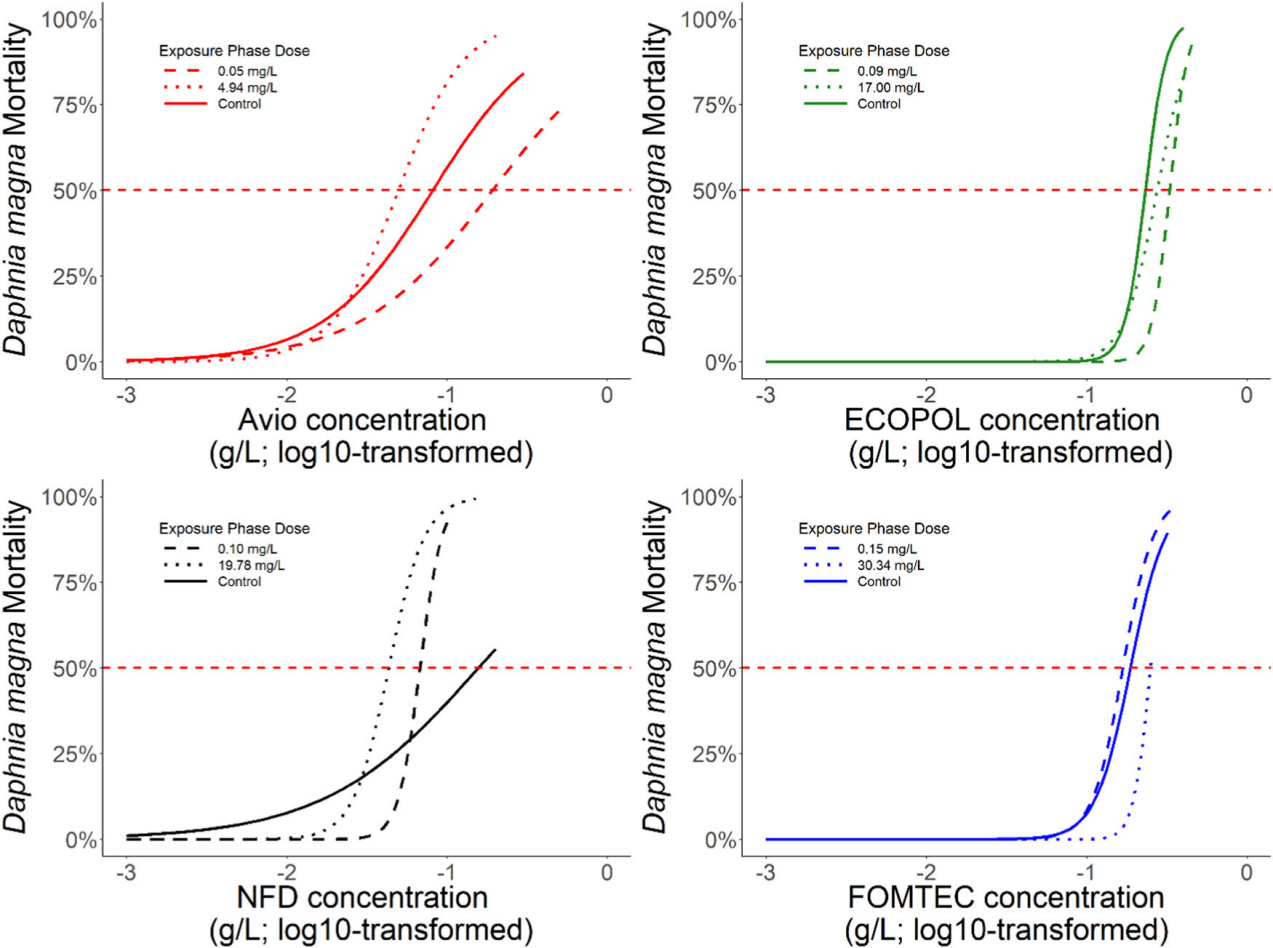
Estimated survival curves of *Daphnia magna* following a 48-hr lethal concentration test using Avio Green, ECOPOL A, NFD 20-391, and FOMTEC Enviro. The red dotted line on each graph denotes 50% mortality. Lines represent the original chronic exposure concentration for each population prior to the acute toxicity tests

**Fig. 3:**
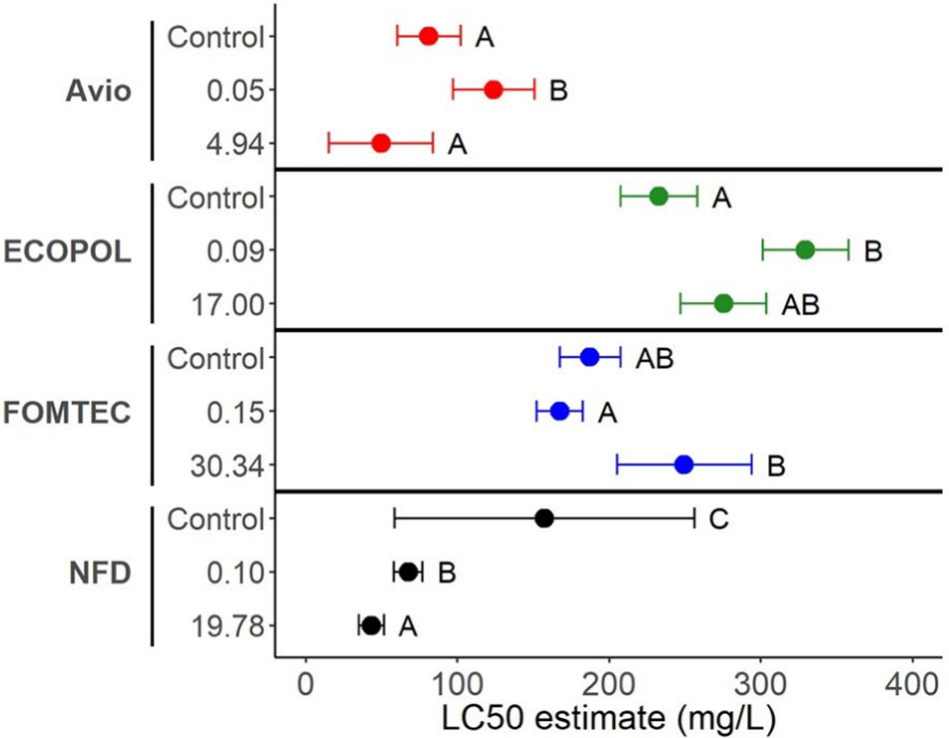
Estimated lethal concentration value (LC_50_) for each *Daphnia magna* population following 84-d of exposure. The original chronic exposure concentration for each population is shown on the y-axis. Data represent estimated mean value with 95% confidence intervals. Simplified results for LC_50_ comparisons following ratio test analyses are provided as letter groups, denoting a significant difference between populations within formulations (α = 0.05)

Avio Green LC_50_ values for *D. magna* populations from the control, 0.0533 and 4.96 mg/L concentrations were 81.3, 124, and 49.7 mg/L, respectively (Fig. 2A, Table 2). The increase in tolerance following exposure to the Avio Green 0.0533 mg/L concentration was significantly different from the tolerance in both the control population (*p* = 0.014) and the 4.96 mg/L population (*p* = 0.002), which did not differ from each other (*p* = 0.095; Fig. 3).

**Table 2:** Lethal concentration values (LC_10_, LC_50_, and LC_90_) for *Daphnia magna* populations exposed to different concentrations of one of seven formulations over 84 days

Formulation	Dose (mg/L)	LC_10_	LC_50_	LC_90_	Ratio test
Avio Green	0.0	14.5 (2.2–28.6)	81.3 (51.2–120.0)	456 (247–2240)	A
	0.0494	48.8 (23.5–66.1)	124 (101–165)	315 (215–828)	B
	4.94	17.5 (NA-40.1)	49.7 (NA-86.1)	141 (82.2-NA)	A
ECOPOL A	0.0	168 (130–189)	233 (210–270)	322 (276–475)	A
	0.085	250 (162–287)	329 (288–357)	434 (392–583)	B
	17.0	164 (35.9–211)	275 (218–358)	461 (356–2260)	A,B
FOMTEC Enviro	0.0	108 (50.1–137)	187 (156–223)	325 (258–687)	A,B
	0.152	104 (67–125)	168 (146–193)	269 (224–422)	A
	30.34	193 (NA-225)	250 (200-NA)	323 (262-NA)	B
NFD 20-391	0.0	13.8 (NA-43.5)	157 (53.5-NA)	1790 (247-NA)	C
	0.099	47.9 (28.1–56.8)	67.7 (57.3–80.7)	95.5 (80.3–167)	B
	19.78	24.7 (2.2–36.5)	43.2 (20.6–62.5)	75.6 (54.5–383)	A
NRL 502W	0.0	804 (NA-1100)	1260 (NA)	1970 (1410-NA)	A
	7.54	992 (470–1160)	1450 (1290–1730)	2120 (1760–5190)	A
	150.7	1440 (915–1170)	2150 (1890–2460)	3220 (2730–5080)	A
Solberg Re-Healing	0.0	98.9 (NA-197)	507 (345-NA)	2600 (845-NA)	A
	0.114	136 (87–171)	314 (271–380)	728 (545–1310)	A
	88.2	150 (54.6–198)	360 (300–592)	864 (549–5340)	A
Buckeye Platinum	0.0	964 (637–1170)	1580 (1350–1780)	2580 (2210–3490)	A
	2.53	1140 (55.9–1760)	2240 (968–2960)	4390 (3240–25,900)	A
	252.6	1010 (627–1280)	2030 (1710–2310)	4070 (3370–5800)	A
	505.1	1740 (645–2210)	2710 (2040–3090)	4230 (3590–7710)	A

The dose corresponds to the 84-d chronic exposure experiment. Data represent the calculated lethal concentration value (mg/L) and the 95% upper and lower confidence limits in parentheses. Estimates for the upper and lower confidence limits are included where calculated. Simplified results for LC_50_ comparisons following ratio test analyses are provided as letter groups, denoting a significant difference between populations within formulations (α = 0.05).

ECOPOL A LC_50_ values for *D. magna* populations from the control, 0.009, and 17.02 mg/L concentrations were 233, 329, and 275 mg/L, respectively (Fig. 2B, Table 2). After comparing the LC_50_ values using a ratio test, we found that the tolerance of *D. magna* exposed to the 0.009 mg/L concentration was increased as compared to the control (Fig. 3). In contrast, the tolerance of *D. magna* exposed to the 17.02 mg/L concentration of ECOPOL A did not differ from those in the control treatment (*p* = 0.190) or the 0.009 mg/L concentration (*p* = 0.170).

NFD 20-391 LC_50_ values for *D. magna* populations from the control, 0.1112, and 19.77 mg/L concentrations were 157, 67.7, and 43.2 mg/L, respectively (Fig. 2C, Table 2). Relative to the control, tolerance was lower following exposure to both concentrations of NFD 20-391 (*p* ≤ 0.037; Fig. 3). Moreover, tolerance was lower in the 19.77 mg/L treatment compared to the 0.1112 mg/L treatment (*p* < 0.001*;* Fig. 3).

While exposure to the formulation FOMTEC Enviro resulted in a marginal change of tolerance from the control population (*p* ≥ 0.089), tolerance of *D. magna* exposed to the 0.1542 mg/L concentration was significantly higher than those exposed to the 30.33 mg/L concentration (*p* = 0.014; Fig. 2D, Table 2). Thus, we observed increased tolerance between populations exposed to FOMTEC Enviro (Fig. 3).

## Discussion

As PFAS-containing AFFF are phased out, there is a need to assess the ecotoxicity of F3 alternatives prior to their widespread adoption for fire suppression activities. After 84-d of exposure to the formulations, we observed changes in tolerance among *D. magna* populations exposed to three of the seven formulations as compared to the tolerance of unexposed populations. We also found marginal changes in a fourth formulation. Evolutionary theory suggests that a change in contaminant tolerance is the result of selection on heritable traits that confer tolerance over multiple generations (Hairston et al. 2001). With a generation time between seven and ten days, we estimate that approximately eight to twelve generations of *D. magna* were exposed to the formulations over the 84-day experiment. Collectively, our results demonstrate that chronic exposure to some F3 formulations can influence the evolution of tolerance in *D. magna* in a relatively short time. Additionally, as previous studies have shown, the response of zooplankton to environmental contamination can have cascading effects on aquatic communities and ecosystems (Bendis and Relyea, 2016; Relyea and Diecks, 2008). Thus, zooplankton are an ideal group for characterizing the evolutionary ecological effects that novel F3 formulations might have on the environment.

This is the first study to find that multi-generational exposure to some F3 formulations (i.e. Avio Green and ECOPOL A) can lead to increased tolerance. Previous research has documented tolerance shifts among zooplankton species following chronic exposure to other contaminants, such as metals (Ward and Robinson, 2005), road salts (Coldsnow et al. 2017; Hintz et al. 2019), increased salinity (Latta et al. 2012), and pesticides (Jansen et al. 2015; Wuerthner et al. 2019). Such increases in tolerance have occurred through changes in the differential expression of genes involved with metabolic pathways in response to thermal stress (Yampolsky et al. 2014), the biosynthesis of osmoprotectants in saline environments (Latta et al. 2012), and upregulation of detoxification genes and compositional shifts in the microbiome following insecticide exposure (Janssens et al. 2022). As we did not investigate the underlying mechanism of evolved tolerance, future research should employ an “omics” approach to identify genes used for detoxification and metabolism of toxic constituents. Additionally, future studies should evaluate if the observed responses persist for longer than three generations.

While evolutionary theory predicts selection on traits that confer increased tolerance as sensitive individuals are lost in exposed populations, this is not always the case (Brady, 2017; Rogalski, 2017). For *D. magna* populations exposed to NFD 20-391, we observed a dose-dependent increase in sensitivity following exposure. Previous research has documented apparently maladaptive evolved responses in zooplankton exposed to pesticides (Jansen et al. 2015) and metals (Rogalski, 2017), as well as in amphibians exposed to increased salinity (Brady, 2017). Additionally, one study found that the toxicity of some PFAS (PFBS and FBSA) intensifies through multigeneration exposure (Xie et al. 2025). The increased sensitivity to NFD is at least superficially similar to the transgenerational effects of these PFAS, which are the class of chemicals that comprise the key ingredients in AFFF that F3 alternatives are replacing. More research is needed to elucidate the mechanism for the apparent maladaptive response in *D. magna* populations exposed to NFD 20-391, and if such selection has also resulted in tradeoffs with life history traits.

Understanding the factors that influence evolved responses to contaminants is vital to predicting outcomes in natural populations. Following an 84-d chronic exposure to Avio Green and ECOPOL A, we observed increased tolerance in *D. magna* populations exposed to the lowest concentration of each formulated product (0.049 and 0.085 mg/L, respectively). Jones et al. (2022) found Avio Green and ECOPOL A to be the two most acutely toxic formulations to *D. magna* (Avio LC_50, 2-d_ = 17.3 mg/L, ECOPOL LC_50, 2-d_ = 19.1 mg/L). Thus, these findings align with evolutionary theory—prolonged exposure to acutely toxic substances shaped the selection of heritable traits that conferred increased tolerance. However, we did not observe a dose-dependent response to each formulation as exposure to higher concentrations did not result in a stepwise increase in tolerance. Non-monotonic responses following exposure to contaminants such as metals (Kimberly and Salice, 2015), pesticides (Liess et al. 2019), and endocrine disrupting chemicals (Lagarde et al. 2015; Vandenberg, 2014; Vandenberg et al. 2012) can occur through cytotoxic effects influenced by gene expression, receptor desensitization, or negative feedback loops. As the current study did not investigate the potential effects of individual constituents in each formulation, future research should aim to identify and examine constituents of concern that might cause such effects at lower concentrations. Additionally, future research that investigates F3 alternative exposure should examine the effects of application frequency and extended exposure (i.e., > 84 d) in populations, as exposure to less toxic F3 alternatives might require more frequent applications or prolonged exposures before evolved responses are observed. Future research should also investigate if these tolerance shifts are lost in greater than 3 generations following exposure, and if so, at what rate. Studies should also confirm the molecular mechanisms behind the observed responses by measuring genetic and epigenetic endpoints. Heritable maternally transferred epigenetics is a growing area of interest in the *Daphnia* model system and likely play a role in the responses observed in this study (Agrelius and Dudycha, 2025).

Additional factors might also influence the evolution of increased tolerance to contaminants. For instance, we did not control for *D. magna* density or monitor sexual reproduction (i.e., genetic crossover) during the 84-day chronic exposure phase of the study. Furthermore, interspecific interactions with amphibians could have influenced evolved responses of *D. magna* populations (Relyea and Hoverman, 2006). Given the opportunistic nature of our experiment, future research should also control for possible co-exposure factors as well as identify important clonal lineages, allele frequency changes, and generational changes in tolerance using a standardized population study (*sensu* Vedamanikam, 2009).

### Conclusion

We observed changes in the tolerance of *D. magna* populations exposed for 84 days to sublethal concentrations of F3 alternatives. These responses have the potential to influence the persistence of zooplankton in natural populations. Our results provide insight into the potential community-level effects (i.e., trophic cascades, indirect effects) that might occur following chronic formulation exposure (Bendis and Relyea, 2016; Relyea and Diecks, 2008). Zooplankton provide key ecological services via consumption of phytoplankton and act as crucial prey items to higher-level consumers. Their population dynamics provide an ideal starting place for understanding the long-term ecological effects of novel F3 alternatives in the environment.

## Data Availability

Data will be made publicly available in the Purdue University Research Repository (https://purr.purdue.edu/), searchable using the article title.
